# Quality of sleep among healthcare workers treating patients with coronavirus disease-19

**DOI:** 10.1192/j.eurpsy.2023.1639

**Published:** 2023-07-19

**Authors:** A. Omrane, I. Touil, E. Sghaier, O. Jaoued, J. Chelly, F. EL Arbi, M. Fkih Hassen, S. Mergheli, J. Knani, T. Khalfallah

**Affiliations:** 1 Occupational Medicine and Ergonomics; 2 Department of Pulmonology; 3 Department of Emergency; 4 Department of Medical intensive care; 5 Department of infectious diseases, Faculty of Medicine of Monastir Tunisia, Monastir, Tunisia

## Abstract

**Introduction:**

Since the declaration of the first Covid-19 case on December 08th ,2019, and to curb the spread of this pandemic, each country and notably Tunisia, has implemented a preventive strategy dominated by general lockdowns in accordance with social distancing and basic hygiene measures. These measures were not applicable in the health care sector as health care workers are at the forefront in the fight against COVID-19. This condition affects not only their physical health caused by elevated workload, but also their mental health causing anxiety, fear, and depression. Previous studies have reported that health care professionals feel stigmatized, experience high levels of anxiety and symptoms of depression, and have sleep problems. Impaired Quality of Sleep (QoS) can affect their efficiency in providing medical services and adequate psychological support for patients suffering from COVID-19.

**Objectives:**

To evaluate the QoS among health care professionals treating patients with COVID-19 and quantifying the symptoms of depression and levels of anxiety.

**Methods:**

A cross-sectional study was conducted in 75 health care professionals matched by age and sex working in public hospital Taher Sfar Mahdia. The study was based in a self administred, French language questionnaire containing three validated questionnaires: 7-item Generalized Anxiety Disorder (GAD-7) Scale, 9-items Patient Health Questionnaire (PHQ-9) Scale, Pittsburgh Sleep Quality Index (PSQI) and additional survey constructed for the purpose of the study.

**Results:**

Healthcare professional treating COVID-19 patients (Group I) group was predominately females mean aged of 32.67±7.04. The health professionals treating COVID-19 patients had poorer Quality of Sleep; Pittsburgh score 10.6 ± 742 vs 7.89 ± 6.14 in the group not treating COVID-19 patients ( p=0.001). Levels of anxiety and depression were significantly higher in the group I (respectively p=0.005 and 0.03). Multiple linear regression analysis revealed that higher scores on GAD (beta = .809, p < .01) and the lower one was the number of persons in charge (beta = –0.632; p < .01) were independent predictors of a poorer quality of sleep

**Conclusions:**

This study has revealed the heavy mental health burden health care professionals treating infected patients in Tunisia during the COVID-19 pandemic are exposed to. Providing early psychological support and a psychologically safe environment for these healthcare workers may alleviate their stress and, consequently, ameliorate their QoS. More attention should be devoted to their quality of sleep and work schedules. In many countries, online training, telehealth supports, behavioral group therapy, cognitive behavioral therapy, and mindfulness-based therapy have been deployed for frontier Healthcare workers and have proven effective in such circumstances.

**Disclosure of Interest:**

None Declared

